# Induced Pluripotent Stem Cells as a Possible Approach for Exploring the Pathophysiology of Polycystic Ovary Syndrome (PCOS)

**DOI:** 10.1007/s12015-023-10627-w

**Published:** 2023-09-28

**Authors:** Masuma Khatun, Karolina Lundin, Florence Naillat, Liisa Loog, Ulla Saarela, Timo Tuuri, Andres Salumets, Terhi T. Piltonen, Juha S. Tapanainen

**Affiliations:** 1 Department of Obstetrics and Gynecology, University of Helsinki, Helsinki University Central Hospital, Haartmaninkatu 8, Helsinki, 00029 HUS Finland; 2 Faculty of Biochemistry and Molecular Medicine, University of Oulu, Oulu, Finland; 3 Institute of Genomics, University of Tartu, Tartu, 51010 Estonia; 4 Department of Genetics, University of Cambridge, Cambridge, CB2 3EH UK; 5 Department of Obstetrics and Gynecology, Research Unit of Clinical Medicine, Medical Research Center, Oulu University Hospital, University of Oulu, Oulu, Finland; 6 Department of Obstetrics and Gynecology, Institute of Clinical Medicine, University of Tartu, Tartu, 50406 Estonia; 7 Competence Centre of Health Technologies, Tartu, 50411 Estonia; 8 Division of Obstetrics and Gynecology, Department of Clinical Science, Intervention and Technology, Karolinska Institutet and Karolinska University Hospital, Huddinge, Stockholm, 14186 Sweden; 9 Department of Obstetrics and Gynecology, HFR – Cantonal Hospital of Fribourg and University of Fribourg, Fribourg, Switzerland

**Keywords:** Polycystic ovary syndrome, Embryonic stem cell, Induced pluripotent stem cell, Genome wide association study, Polygenic risk score

## Abstract

Polycystic ovary syndrome (PCOS) is the most prevalent endocrine condition among women with pleiotropic sequelae possessing reproductive, metabolic, and psychological characteristics. Although the exact origin of PCOS is elusive, it is known to be a complex multigenic disorder with a genetic, epigenetic, and environmental background. However, the pathogenesis of PCOS, and the role of genetic variants in increasing the risk of the condition, are still unknown due to the lack of an appropriate study model. Since the debut of induced pluripotent stem cell (iPSC) technology, the ability of reprogrammed somatic cells to self-renew and their potential for multidirectional differentiation have made them excellent tools to study different disease mechanisms. Recently, researchers have succeeded in establishing human in vitro PCOS disease models utilizing iPSC lines from heterogeneous PCOS patient groups (iPSC_PCOS_). The current review sets out to summarize, for the first time, our current knowledge of the implications and challenges of iPSC technology in comprehending PCOS pathogenesis and tissue-specific disease mechanisms. Additionally, we suggest that the analysis of polygenic risk prediction based on genome-wide association studies (GWAS) could, theoretically, be utilized when creating iPSC lines as an additional research tool to identify women who are genetically susceptible to PCOS. Taken together, iPSC_PCOS_ may provide a new paradigm for the exploration of PCOS tissue-specific disease mechanisms.

**Graphical Abstract:**

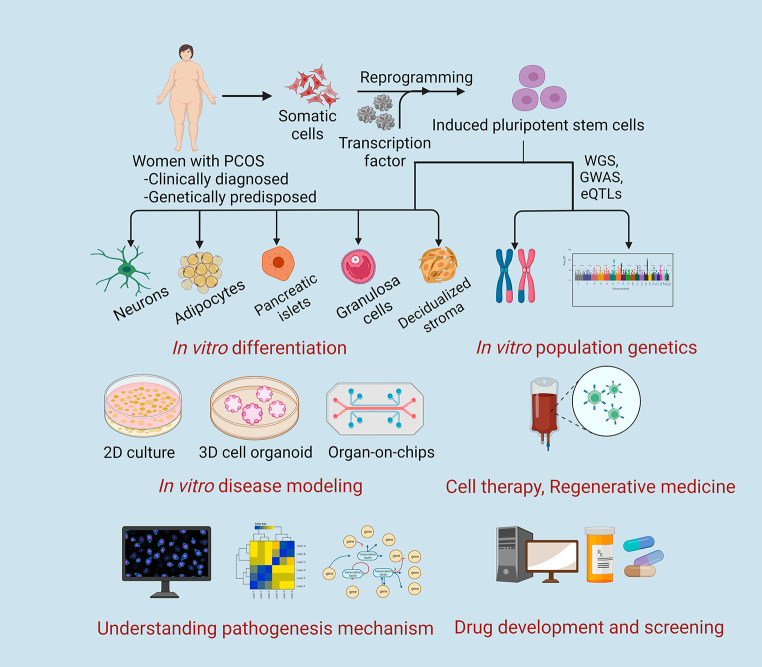

## Introduction

Polycystic ovary syndrome (PCOS) is a complex endocrine and metabolic condition affecting 4–20% of reproductive-aged women depending on demographic and diagnostic criteria [1, 2]. The condition has vast multimorbidity [3] and is characterized by irregular menstrual cycles, elevated levels of androgens, insulin resistance (IR), ovarian dysfunction, chronic inflammation, impaired glucose metabolism, dyslipidemia, an increased risk of cardiovascular disease (CVD), and mental distress [4–6]. The International PCOS guideline recommends Rotterdam criteria for PCOS diagnosis [7], where at least two of three of the following features should be present: irregular menstrual cycles, clinical or biochemical hyperandrogenism (HA), and polycystic ovarian morphology (PCOM) in ultrasound [8].

PCOS has also been shown to inflict a significant socio-economic burden [9, 10]. Moreover, the syndrome is an independent risk factor for psychological distress and a lower quality of life [11, 12]. As several key questions relating to the etiology of PCOS, the criteria used to diagnose the syndrome, and its optimal treatment practices remain unresolved, the investigation of PCOS pathogenesis and the thorough delineation of its underlying mechanisms constitute an active area of current research [13, 14].

## Pathophysiological Features of PCOS

### Hyperandrogenism (HA) and Insulin Resistance (IR)

**Fig. 1:**
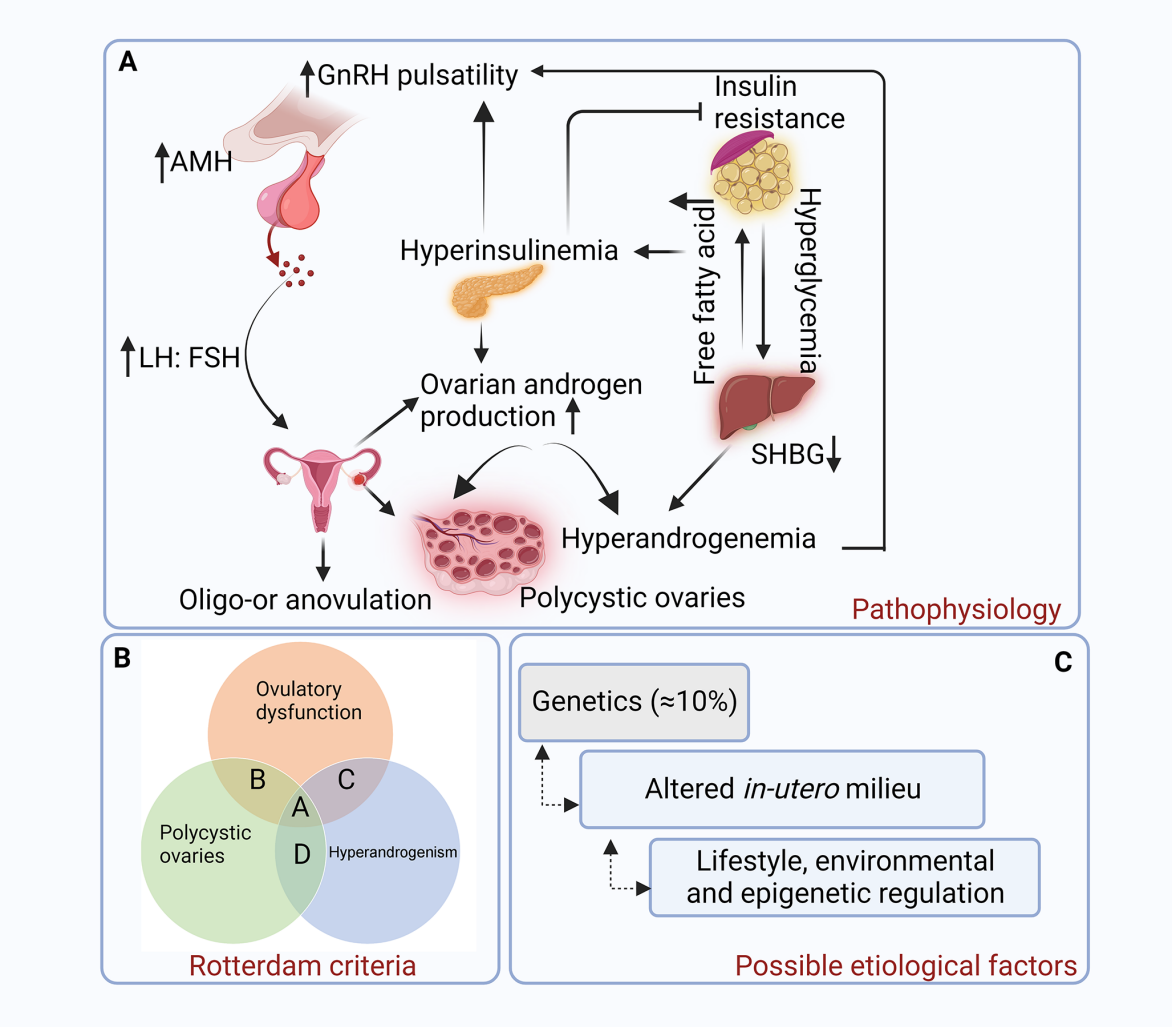
(**a) Pathophysiology of polycystic ovarian syndrome (PCOS).** Women with PCOS have impaired neuronal circuits in the brain, resulting in increased GnRH pulsatility, which in turn causes the hypersecretion of LH and subsequent HA from the ovarian theca cells. Follicular maturation is inhibited by excessive AMH secretion, which downregulates FSH action. This results in follicular arrest, polycystic ovarian morphology, and ovulatory dysfunction. The high AMH concentration also stimulates GnRH neuron activity and directly drives the GnRH-dependent production of LH, which may further promote ovarian HA. Predisposition to excessive ovarian androgen production is the primary defect in PCOS. Additionally, IR leads to hyperinsulinemia, which elevates GnRH release and increases androgen production in ovarian theca cells while suppressing SHBG production, causing HA; **(b) Rotterdam criteria diagnostic consensus 2004**. Includes at least two of three features: phenotype A (polycystic ovaries + ovulatory dysfunction + excessive androgen/HA), phenotype B (ovulatory dysfunction + HA), phenotype C (polycystic ovaries + HA), and phenotype D (polycystic ovaries + ovulatory dysfunction); **(c) Possible etiological factors in PCOS pathogenesis.** GnRH, gonadotrophin-releasing hormone, LH, luteinizing hormone, HA, hyperandrogenism, AMH, anti-Müllerian hormone, FSH, follicle-stimulating hormone, IR, insulin resistance, SHBG, sex hormone-binding globulin. Solid line arrows are used to indicate increase or decrease. Dashed line arrows are used to indicate influence. Created with BioRender.com

The majority of women with PCOS (~ 60%) diagnosed via the Rotterdam criteria exhibit HA [15, 16]. In addition, obesity, an independent risk factor for PCOS, also increases HA and exacerbates many metabolic and reproductive disorders, including impaired insulin sensitivity and secretion [17, 18]. IR and hyperinsulinemia lead to decreased levels of sex hormone-binding globulin (SHBG), which in turn cause an increase in free androgens and adverse metabolic profiles [19, 20]. Collectively, although the underlying pathogenic mechanisms of PCOS are still unknown, obesity and IR aggravate the symptoms of HA, forming a vicious cycle that promotes the development of PCOS (Fig. 1) [21, 22].

### Neuroendocrine Dysfunction and Disrupted Ovarian Folliculogenesis

The pulsatile release of gonadotrophin-releasing hormone (GnRH) stimulates the secretion of luteinizing hormone (LH) and follicle-stimulating hormone (FSH) from the anterior part of the pituitary gland, which in turn regulates ovarian steroid production [23]. Interestingly, women with PCOS have been found to have an elevated LH to FSH ratio independent of obesity due to an increased frequency of GnRH pulses, suggesting hypothalamic neuronal dysregulation [24, 25]. Increased levels of LH stimulate androgen secretion from the ovarian theca cells, whereas low levels of FSH decelerate follicle development, leading to typical follicular arrest and PCOM in affected women [26, 27]. Furthermore, due to the increased number of small and preantral follicles in PCOS [28, 29], anti-Müllerian hormone (AMH) secretion is considerably higher in women with the syndrome than in ovulatory women, causing defective folliculogenesis through the inhibition of aromatase activity and FSH action [30, 31]. All in all, dysfunctional neurocircuits play a crucial role in PCOS pathogenesis (Fig. 1) [32, 33].

### Anovulation and Endometrial Dysfunction

PCOS is the most common cause of anovulatory infertility [34, 35] and increases the risk of endometrial dysfunction [36, 37]. Particularly hyperandrogenic women with PCOS present with the risk of preeclampsia, aberrant trophoblast invasion, shallow placentation, disrupted uterine decidualization, and angiogenesis, which collectively indicate endometrial dysfunction [38–40]. Indeed, endometrium-derived stromal cells from women with PCOS also exhibit altered biological functions related to defective insulin signaling, disrupted cell cycle, altered glucose metabolism, aberrant steroid receptors, progesterone (P4) resistance, with greater risk of compromised stress tolerance, and elevated oncogenic potential [41–45]. Furthermore, HA and IR may increase the risk of miscarriage by disrupting mitochondrial biogenesis, and oxidative stress was evident in the gravid uterus and placenta in a PCOS-like rodent model [46, 47].

### Systemic low-grade Inflammation

Women with PCOS exhibit systemic, low-grade, chronic inflammation, which contributes to an increased risk of coronary heart disease (CHD) and type 2 diabetes mellitus (T2DM) as seen by elevated C-reactive protein (CRP), even irrespective of obesity [48, 49]. In addition to serum CRP, women with PCOS also have higher levels of peripheral lymphocytes, monocytes, eosinophilic granulocytes, tumor necrosis factor (TNF-α, β), adipokines, and interleukins (IL-6, 10, 12, 18, 34) [50, 51]. Particularly, PCOS ovaries have more inflammatory cells and ongoing chronic inflammation in comparison to healthy ovaries [52, 53]. In fact, peripheral B cell count and activity are higher in PCOS-afflicted women [54], which have been proven to be direct modulators of androgen receptor activation and may therefore contribute to PCOS pathogenesis [55].

## Etiology of PCOS

### Genetic Heritability and genome-wide Association Studies (GWAS)

Despite the fact that the clinical symptoms of PCOS usually worsen after the maturation of the hypothalamus-pituitary-ovary axis during puberty, numerous clinical and experimental studies have shown that the syndrome has a strong genetic basis [56–58]. Indeed, studies of familial PCOS have suggested an autosomal dominant inheritance pattern; however, research on the mode of inheritance remains inconclusive [59–61]. According to a twin study, PCOS is a highly heritable condition with a disease correlation of 71% in monozygotic twins, about twice as high as that in dizygotic twins (38%), suggesting a significant genetic component to the disorder [62]. Furthermore, a longitudinal study of daughters of mothers with PCOS who were followed from infancy to postmenarche indicated that the PCOS phenotype (elevated LH, HA, and IR) during the postmenarcheal period contributes to the development of PCOS during adulthood [63]. The presence of a significant genetic component in PCOS etiology is also consistent with the fact that there is an increased prevalence of metabolic disorders, hypertension, and hyperlipidemia in first-degree relatives of women with PCOS [64–66].

Genome-wide association studies (GWAS) have considerably advanced our understanding of the pathophysiology of PCOS by highlighting over 20 loci across the genome that are significantly associated with PCOS in different human populations [67]. Common PCOS-associated variants linked to genes, such as thyroid adenoma-associated protein (THADA), insulin receptor (INSR), follicle-stimulating hormone receptor (FSHR), ERBB4 receptor tyrosine kinase 4 gene (ERBB4), and DENN domain containing 1 A (DENND1A), confer mostly obesity-related metabolic risk, IR, impaired folliculogenesis, and abnormal androgen biosynthesis [68–72]. A recent meta-analysis of GWAS showed that the diagnostic criteria for common genetic variants of PCOS at 13 risk loci were similar, supporting the notion that the different diagnostic criteria do not pinpoint genetically distinct disease subtypes [73]. A review of SNPs and the nearby candidate genes associated with PCOS are listed in Table 1. However, the complex PCOS syndrome cannot be explained by a small number of variants with limited effects on such diverse phenotypes. A key challenge here is, recruiting large representative case-control cohorts with sufficient power, as 75% of women with PCOS go undiagnosed until they are of reproductive age [74]. All things considered, genetic research remains considerably challenging for such a multifactorial disease as PCOS.

**Table 1:** Genome-wide association studies (GWAS) revealing the candidate single nucleotide polymorphism (SNP) and nearby genes for PCOS predisposition

Gene locus	SNPs	Nearest genes	References
2q34	rs7564590	*ERBB4*	^β^ [69]
9q33	rs3945628	DENND1A*	
11p14	rs11031002	*FSHB*	
11q23	rs1672716	*ZBTB16*	
5p15	rs9312937	*MYO10*	
22q12	rs145598156	*CHEK2*	
2q21	rs7563201	THADA*	^β,¥^ [73]
2q.34	rs2178575	*ERBB4*	
5q31.1	rs13164856	*IRF1/RAD50*	
8p32.1	rs804279	*GATA4/NEIL2*	
9p24.1	rs10739076	*PLGRKT*	
9q22.32	rs7864171	C9orf3 *	
9q33.3	rs9696009	DENND1A *	
11p14.1	rs11031005	*ARL14EP/FSHB*	
11q22.1	rs11225154	YAP1 *	
11q23.2	rs1784692	*ZBTB16*	
12q13.2	rs2271194	*ERBB3/RAB5B*	
12q21.2	rs1795379	*KRR1*	
16q12.1	rs8043701	TOX3 *	
20q11.21	rs853854	*MAPRE1*	
2q.34	rs1351592	*ERBB4*	^β^ [224]
11q22.1	rs11225154	YAP1	
2q21	rs7563201	*THADA*	
11p14.1	rs11031006	*FSHB*	
5q31.1	rs13164856	*RAD50*	
12q21.2	rs1275468	*KRR1*	
8p32.1	rs804279	*GATA4, NEIL2*	^β,α^ [225]
9q22.32	rs10993397	*C9orf3*	
11p14.1	rs11031006	*ARL14EP, FSHB*	
8q24.2	rs10505648	*KHDRBS3, LINC02055*	^α^ [226]
12p12.2	rs10841843 rs6487237 rs7485509	*GYS2*	[227]
2p16.3	rs13405728	*LHCGR, STON1-GTF2A1L*	[70]
2p16.3	rs2268361 rs2349415	*FSHR*	
2p21	rs12468394 rs13429458 rs12478601	*THADA*	
9q33.3	rs10818854 rs2479106 rs10986105	*DENND1A*	
9q22.32	rs4385527 rs3802457	*C9orf3*	
11q22.1	rs18974116	*YAP1*	
12q13.2	rs705702	*RAB5B, SUOX*	
12q14.3	rs2272046	*HMGA2*	
16q12.1	rs4784165	*TOX3*	
19p13.3	rs2059807	*INSR*	
20q13.2	rs6022786	*SUMO1P1*	
2p16.3	rs13405728	*LHCGR, STON1-GTF2A1L*	[71]
2p21	rs12468394 rs13429458 rs12478601	*THADA*	
9q33.3	rs10818854 rs2479106 rs10986105	*DENND1A*	

^β^ Studies on European population, ^¥^ Rotterdam and NIH criteria, * Common in both Han Chinese and Europeans, ^α^ NIH criteria

### Environmental Factors and Epigenetic Regulation

Currently, GWAS of direct genetic variants of PCOS have explained only 10% of its heritability, supporting the idea that multiple environmental and lifestyle factors, as well as epigenetic regulation of the genome, may interact in the onset of PCOS [75–79]. Environmental toxins have been linked to an increased risk of PCOS, particularly endocrine-disrupting chemicals (EDCs), such as bisphenol A (BPA), which has been positively correlated with HA [80–82]. The likelihood of ovulatory dysfunction in PCOS-affected women is also increased by smoking, a hypercaloric diet, and exposure to plastics [83]. Epigenetic alterations and both heritable and non-heritable changes in gene expression not affecting underlying DNA sequences, have also been offered as a possible explanation for missing heritability in this complex metabolic disorder [79, 84, 85]. Indeed, recent data suggest that epigenetic alterations play important roles in the development of PCOS [86–88].

### Intergenerational Transmission of PCOS

Despite the fact that clinical symptoms of PCOS do not manifest until adolescence, it is clear that the natural history of the syndrome is rooted in the intrauterine environment through developmental programming. Women with PCOS usually present with a high oocyte yield in in vitro fertilization (IVF), and the quality of oocytes does not appear to be significantly different from that of women without PCOS [89, 90]. However, women with PCOS who are being treated with assisted reproductive technology (ART) often present with unfavorable pregnancy outcomes, possibly via the negative effect of altered ovarian or uterine factors on the competence of oocytes through endocrine/paracrine actions [91, 92]. According to a 2023 study by Risal et al., male offspring of obese and hyperandrogenic mothers with PCOS frequently suffer from obesity and dyslipidemia, suggesting altered metabolic and reproductive profiles across generations [93]. All of these findings strongly support the notion that PCOS is the continuation of a process that begins during intrauterine life [94–97].

Additionally, numerous data from human and animal models demonstrate that fetal exposure to excess androgen causes alterations in developing tissues, leading to the development of PCOS in adulthood [98–100]. Zhang et al. discovered that rats with PCOS that were administered insulin alone or in combination with human chorionic gonadotrophin (hCG) displayed defective uterine PI3K/Akt signaling pathways, which was used as an indicator of the onset of uterine IR [101]. This finding addressed the role of IR and HA in the altered uterine environment caused by PCOS. In addition, AMH appears to be one of the key factors in PCOS pathogenesis through the reprogramming of the fetus and possibly predisposes one to exhibit PCOS traits in adulthood, as pregnant mice administered a high dose of AMH experienced persistent hyperactive GnRH pulsatility that was passed on to female progeny [102, 103].

Considering such diverse etiological factors, a thorough understanding of the pathogenesis of PCOS is essential for developing a tailored treatment plan. Currently, animal models are being extensively used to mimic the pathological characteristics of PCOS in patients [99, 104]. The discrepancy between animal models and human physiology has, however, raised questions regarding the feasibility of using animal models to explore PCOS etiology.

## Embryonic stem cell Research in Women with PCOS

Human embryonic stem cells (hESCs) enable versatile and well-regulated in vitro models to study disease pathophysiology. PCOS-derived hESC (hESC_PCOS_) models could be a rational and effective strategy for not only investigating the pathogenesis of PCOS in its early developmental stage but also for identifying the genetic factors that contribute to the onset of the syndrome. To comprehensively investigate all of these possibilities in the context of PCOS etiology, Li et al. established the first hESC_PCOS_ lines from the inner cell mass (ICM) of blastocyst stage embryos from women with PCOS. According to their findings, hESC_PCOS_ present with abnormal lipid metabolism, one of the vital features of PCOS pathogenesis [105]. Subsequently, the global gene expression data have also revealed that adipocytes differentiated from hESC_PCOS_ possess a substantial number of differentially expressed genes (DEGs) (n = 153; 91 upregulated and 62 downregulated genes) compared to non-PCOS-derived hESC controls (hESC_Ctrl_). Most of the DEGs (e.g., *NR0B2, HSD3B2, TSPAN8, HLA-DRB3*, and *UGT2B28*) were linked to glucose, lipid, and steroid hormone metabolism [105], suggesting an underlying defect in biological functions associated with obesity and IR in PCOS, consistent with other studies [106]. Following prior studies, Zhang et al. successfully established and characterized 10 hESC_PCOS_ lines capable of normal growth, germ layer differentiation, and teratoma formation in vitro, confirming all of the characteristics of hESC_PCOS_ similar to non-PCOS control cells [107]. Despite the significance of hESC research in understanding the etiology of PCOS, it is impossible to create hESC lines for every patient utilizing ART [108, 109]. In addition, studying human embryos is highly restricted, and often prohibited, due to local legal frameworks, further challenging the investigation of the pathology of PCOS at its early developmental stage [110].

## Progression of Induced Pluripotent stem cell (iPSC) Technology in PCOS Research

Given the importance of human pluripotent stem cells (hPSCs) in in vitro research and their wide spectrum of potential applications, breakthrough discoveries in cellular reprogramming *via* induced pluripotent stem cell (iPSC) technologies allow for the creation of patient-derived cells that exhibit all of the genetic features possibly associated with a specific disease [111]. Similar to hESCs, patient-derived iPSCs have the potential to differentiate into a variety of cell types, including neurons [112], hematopoietic cells [113], cardiomyocytes [114], glia cells [115], and pancreatic islets [116]. Furthermore, three-dimensional (3D) “organoid” models of female reproductive tissue, such as the uterus, fallopian tubes, ovaries, and trophoblast, all produced from iPSCs, have recently emerged as a valuable tool for simulating the physiological processes involved in the progression of gynecological diseases in vitro [117–121]. In addition, scientists have recently developed the ability to differentiate human iPSCs into endometrial stromal cells with spontaneous decidualization capabilities, which is one of the key events in the successful implantation process [122, 123]. Research on and potential future therapies for endometriosis, PCOS-driven endometrial dysfunction, early-stage endometrial cancer, and uterine factor infertility can all benefit from these findings, which have ushered in a new era of cell therapy for endometrial disease. Upon general consideration, iPSC generation via cellular reprogramming has been regarded as a major step forward in biological science, providing a potential tool for disease modeling, drug screening, customized treatment, and tissue/organ regeneration free of ethical concerns. However, the iPSC technology has not been widely adopted in the field of PCOS pathophysiology. An overview of the existing data on the use of hPSCs in PCOS research is outlined in Table 2.

**Table 2:** An overview of the key findings and potential limitations of human pluripotent stem cell research in women with PCOS.

Cell line	IVD	Experiment design	Major findings	Limitations	Reference
hESCs	X	Embryos from women with PCOS who underwent IVF	First established hESC_PCOS_ (n = 5)	-Ethical concern -Derived from 0-2PN (arrested) embryos	[105]
hESCs	Adipocyte	Adipocyte differentiation ± insulin, Glucose measurement (24 h) by oxidase method	No difference in glucose consumption during adipocyte differentiation in hESC_PCOS_ (n = 3) vs. hESC_Ctrl_ (n = 1)	-Previous hESCs [105] -Small sample size -Poorly characterized cells	[228]
hESCs	Adipocyte	Microarray	Altered DEGs linked to glycolipid metabolism, and steroid regulation in adipocytes hESC_PCOS_ (n = 3) vs. hESC_Ctrl_ (n = 1)	-Previous hESCs [228] -Small sample size -Low yield (50%) and poorly characterized cells	[106]
hESCs	X	Embryos from women with PCOS who underwent IVF	Comparable differentiation potential hESC_PCOS_ (n = 10) vs. hESC_Ctrl_ (n = 8)	-Ethical concern -No functional studies	• [107]
uEP-iPSCs	Adipocyte	Adipocyte differentiation ± insulin, Glucose measurement (24 h) by oxidase method	Higher glucose consumption during adipocyte differentiation in iPSC_PCOS_ vs. iPSC_Ctrl_ (n = 5)	-Retroviral vector -Low yield and poorly characterized cells	• [136]
dF-iPSCs	X	Microarray, mitochondrial respiration assay by seahorse XF analyzer, metformin effect	-GO analysis: up-regulated DEGs enriched in metabolic-mitochondrial activities; down-regulated DEGs linked to glucose metabolism in iPSC_PCOS_ (n = 3) -Decreased mitochondrial and glycolytic function; increased mitochondrial copy and biogenesis in iPSC_PCOS_ (n = 3) -Minor metformin effect on mitochondrial respiration and glycolysis in iPSC_PCOS_ vs. iPSC_Ctrl_ (n = 3) -Validation of DEGs in primary GCs from PCOS, Ctrl (n = 5)	-Lentiviral vector -Small sample size	• [164]
dF-iPSCs	NSCs	Microarray, ELISA for T/E2 measurement for iPSC_PCOS/Ctrl,_ mitochondrial respiration assay for primary GCs and NSCs	-GO analysis: down-regulated DEGs linked to neurogenesis; up-regulated DEGs linked to neural crest development and P4 action in iPSC_PCOS_ vs. iPSC_Ctrl_ (n = 3) -Higher T level in iPSC_PCOS_ (n = 3) -Decreased respiration in primary GCs and iPSCs in PCOS vs. Ctrl (n = 3) -Decreased respiration in NSCs from iPSC_PCOS_ (n = 3)	-Lentiviral vector -Small sample size	• [192]
dF-iPSCs	GCs	Aromatase (CYP19A1) Activity assay, Whole-genomic DNA methylation profile by Illumina 850 K MethylationEPIC BeadChip	-Higher E2 secretion in GCs from iPSC_PCOS_ (n = 2) -Hyperactive CREB in GCs (PCOS vs. Ctrl, n = 2) and primary GCs in PCOS (n = 11) vs. Ctrl (n = 4) -Higher CBP expression in the iPSC-derived (PCOS, Ctrl, n = 2) and primary GCs in PCOS (n = 5) vs. Ctrl (n = 4)	-Episomal vector -Small sample size -Low yield and poorly characterized GCs	• [165]
PBMC-iPSCs	X	X	Established iPSC_PCOS_ (AMUFAHi002-A) (n = 1)	-Episomal vector -No Ctrl and functional study	• [229]

*PCOS women screened by Rotterdam 2003 criteria [7], IVD, in vitro differentiation, X, no differentiation, hESCs, human embryonic stem cells, iPSCs, induced pluripotent stem cells, IVF, in vitro fertilization, PN, pronuclear, ± with and/or without, Ctrl, non-PCOS control women, uEP, urine epithelium, dF, dermal fibroblasts, PBMC, peripheral blood mononuclear cell, GO, gene ontology, DEGs, differentially expressed genes, T, testosterone, E2, estradiol, P4, progesterone, NSCs, neural stem cells, GCs, granulosa cells, CREB, cAMP response element-binding element, CBP, CREB-binding protein

### Adipocyte Dysfunction

Mounting evidence suggests that, overall, obesity worsens the severity of PCOS and anovulation-related disorders [124, 125]. Furthermore, obese women with PCOS are more prone to abdominal visceral adiposity, independent of their body mass index (BMI) [126, 127]. In fact, HA inhibits early-stage adipogenesis, reduces insulin-stimulated glucose uptake, and promotes lipid storage [128–130]. Insulin, in turn, promotes visceral fat deposition by amplifying androgen synthesis followed by multiple comorbidities by ovarian theca cells [131–133]. Moreover, obesity and HA have a significant impact on the adipokine secretion profile and unfavorable inflammatory profile in these women [134, 135].

To study obesity-related incidences at the developmental stage, Yang S. et al. successfully reprogrammed PCOS-derived urine epithelial cells into iPSCs and subsequently differentiated the cells into adipocytes. In their study, iPSC_PCOS_ presented with a greater capacity for glucose consumption throughout adipocyte differentiation along with a lower insulin response in vitro compared to iPSC_Ctrl_ [136]. These findings are indicative of defective adipocyte function and IR, which have been shown to be common traits in women with PCOS, especially those who are obese and hyperandrogenic [134, 137–141]. Additionally, women with PCOS exhibit increased steroidogenesis in adipocytes, which is considered to be an important factor in the onset and maintenance of PCOS [142, 143]. Therefore, to better understand the altered metabolic dysregulation in this context, the dynamics of adipokine secretion in relation to obesity during adipocyte development require special attention. All in all, a comprehensive study with a large sample size of BMI-classified women with PCOS is required to more fully understand the mechanism underlying adipocyte differentiation in an iPSC_PCOS_ model.

### Granulosa cell (GC) Dysfunction and Altered DNA Methylation

Granulosa cells (GCs), an important ovarian somatic component, regulate follicular development and proliferation, produce sex hormones, and secrete other growth factors [144]. In PCOS, altered GC functions contribute to abnormal folliculogenesis, including decreased apoptosis, defective proliferation, abnormal hypersensitivity to FSH stimulation, and altered steroidogenesis [28, 145–147]. A multi-omics investigation further confirmed that DEGs involved in steroid production and metabolic signaling cluster differently in PCOS-derived GCs than in normal GCs [148–150]. Indeed, an altered oocyte microenvironment with perturbed gene expression in both human and murine PCOS-derived GCs has been demonstrated [151, 152]. A recent study further demonstrated that, independent of IR, the GCs of women with PCOS exhibit metabolic distress and elevated DEGs in the endoplasmic reticulum and mitochondria compared to women without PCOS [153]. However, these studies used GCs isolated from women undergoing IVF treatment after ovarian stimulation, which are different from in vivo conditioned human-derived ovarian GCs [154–157]. The precise pathogenic contribution of GCs to PCOS development *in utero* thus remains unclear due to the lack of an appropriate research model. Moreover, it is not clear whether differentiated GCs-derived from iPSC lines can mimic in vivo condition.

To investigate the GC profile in women with PCOS, Min Z. et al. validated their microarray data from undifferentiated iPSC_PCOS_ with data from the primary GCs of PCOS vs. non-PCOS control patients. They found that ovarian folliculogenesis-related genes, such as *FBP1, IL-18*, and *SOAT1*, are significantly upregulated in iPSC_PCOS_, which was consistent with their data from primary PCOS-derived GCs compared to non-PCOS-derived GCs. Indeed, *FBP1*, a key regulator of oocyte maturation, the insulin signaling pathway, and glucose homeostasis during early embryogenesis, has been found to be linked to abnormal development of murine ovarian follicles when administered with high testosterone [158]. In line with this, transcriptome data from cumulus cells derived from obese women with PCOS but without IR who were undergoing IVF treatment showed a higher expression of *FBP1*, suggesting an impaired follicular environment in these women even in the absence of IR [159]. In contrast, increased *IL-18*, a pro-inflammatory cytokine secreted by ovarian GCs, has also been linked to the alteration of the follicular microenvironment in women with PCOS [160–162]. Furthermore, the distinct expression of *SOAT1*, a key regulator of adrenal steroidogenesis, has been associated with abnormal follicular development in a rodent PCOS model [163]. Taken together, the data suggest that impaired folliculogenesis is present in women with PCOS; however, further studies of iPSC-derived GCs that mimic in vivo conditions are needed to confirm this hypothesis [164].

In the context of epigenetic modifications, the DNA methylation profile of cells in women with PCOS at its developmental stages is unknown. To date, only one study by Huang et al. has addressed this issue. The team reported that the whole-genomic DNA methylation pattern is significantly different in the primary GCs of PCOS patients and the differentiated GCs of iPSC_PCOS_ with hypo- and hyper-methylated genes compared to non-PCOS subjects [165]. According to their methylomic enrichment pathway analysis, a total of 472 differentially methylated region (DMR)-located genes in the primary GCs and 3,682 DMR-located genes in the differentiated GCs of iPSC_PCOS_ were mostly related to protein kinase C (PKC), protein kinase A (PKA), and phosphatidylinositol-3 kinase (PI3K) signaling. These were linked to many regulatory pathways in the MetaCore analytic database, such as the thromboxane A2 signaling pathway, the cAMP response element-binding protein (CREB) signaling pathway, the nociception receptor signaling pathway, oxidative stress, and proinsulin C-peptide signaling. In the PCOS group, the hyperactive CREB signaling pathway, which is a critical sensor for both hormonal and metabolic signals, was found to be consistent in both primary and iPSC-derived GCs. Furthermore, Huang et al. were able to validate the hyperactive CREB signaling data by confirming the presence of significantly higher levels of CREB-binding protein (CBP) in both iPSC-derived and primary GCs in the PCOS group compared to the non-PCOS group [165]. Indeed, estrogen (E2)-induced chronic CREB signaling pathway activation with aberrant aromatase activity and metabolic disorders have been found in in vitro studies of mature GCs from women with PCOS [166–168].

Based on the results from iPSC_PCOS_, there is no substantial difference in the pluripotency and differentiation potential between those with and without PCOS, despite the existence of pathogenic features. Even after somatic cell reprogramming and differentiation, ovarian GCs derived from iPSC_PCOS_ retain most of their common properties and functions compared to those from women without PCOS. However, Huang reported that GCs derived from iPSC_PCOS_ showed an increased expression of GC-specific markers, including AMH, AMH receptor 2 (AMHR2), and FSHR, as compared to women without PCOS. Expectedly, these data are consistent with earlier findings that revealed intrinsically abnormal folliculogenesis in women with PCOS [169, 170]. This could be due to the fact that particular GC-associated functional genes are expressed more frequently in both early differentiated cells and adult cells in women with PCOS, supporting the idea that the onset of PCOS occurs at an early developmental stage. The common overexpressed genes found in both iPSC-derived GCs and adult GCs in both studies indicate that GCs in PCOS can be responsible for hormonal dysregulation already during the early developmental stage rather than being a result of environmental or behavioral changes. On the other hand, the assessment of epigenetic memory via cellular reprogramming is not so straightforward, as cellular resetting methods can also reset genomic methylation using a different mechanism and kinetics from those seen in vivo [171, 172]. Although iPSCs were shown to possess, to some extent, various epigenetic and transcriptional differences compared to hESCs, these dissimilarities do not appear to have a functional impact on cellular differentiation in PCOS vs. non-PCOS controls [173]. However, further studies with relatively bigger sample sizes are needed to draw firm conclusions about these observations.

### Mitochondrial Biogenesis and Metformin Effect

It is well established that mitochondrial malfunction at the cellular level can disrupt systemic metabolic homeostasis [174, 175]. In recent studies, increased oxidative stress has been linked to the onset and progression of PCOS, thereby strengthening the association between mitochondrial dysfunction and PCOS [47, 176, 177]. As GCs rely on mitochondrial respiration and glycolysis for energy, any anomalies in this synergy during early follicular development can result in metabolic failure, impaired glucose metabolism, and persistent inflammation [59]. Global gene expression data presented by Min Z. et al. revealed that out of a total of 2,904 DEGs, 1,416 were upregulated in iPSC_PCOS_ (Fold Change (FC) > 30; *IFI16, CAPN6, LAMA4, IL18, FOLH1, TBX5, FBP1, AGL*, and *KIAA1324*) and were enriched in metabolic processes and mitochondrial functions specifically linked to the tricarboxylic acid cycle, respiratory electron transport chain, and glycogenolysis compared to iPSC_Ctrl_ [164]. In contrast, the top 10 significantly downregulated genes, such as *FN1, NTS, CER1, SPP1, SLC7A3, ZFP42, HAS2, PTPRZ1*, and *CDH1*, were found to be associated with cell communication, glucose transport, cytokine activity, neurogenesis, calcium-phosphate binding, and endocrine metabolism [164].

The mitochondrial respiration and glycolytic function of iPSC_PCOS_ were significantly impaired, indicating a potential mitochondrial defect at the developmental stage, similar to the findings for GCs from women with PCOS undergoing IVF treatment and in the cumulus cells of diabetic mice [178, 179]. When comparing iPSC_PCOS_ to iPSC_Ctrl_, an unexpected increase in the number of mitochondrial DNA (mtDNA) copies was discovered [164]. Interestingly, the expression levels of mitochondrial biogenesis-related genes (*PGC-1α*, *TFAM*, and *NRF1*) were significantly higher, which is commensurate with increased mtDNA copies, confirming increased mitochondrial biogenesis in women with PCOS [164]. However, the correlation between increased mtDNA content or biogenesis with disease condition is ambiguous as numerous factors are involved in the transcriptional and post-transcriptional regulation of gene expression at the genetic and epigenetic levels [180–182]. One possible interpretation of the findings could be that mitochondrial biogenesis is increased to compensate for mitochondrial malfunction in iPSC_PCOS_. As a compensatory response, the aberrant metabolic state of PCOS necessitates more energy to advance the synthesis of mitochondria. Furthermore, the reduced expression of glucose transporters (GLUT1 and GLUT3) and concomitant mitochondrial dysfunction may be linked to IR in iPSC_PCOS_, in line with other studies [164, 183, 184]. However, there have been conflicting findings on mitochondrial oxidative phosphorylation (OXPHOS), mostly focusing on skeletal muscles and adipose tissues in women with PCOS [47]. These contradictory results could also be the result of heterogeneous clinical manifestations in women with PCOS along with variable experimental designs, methods, and sample sizes. Therefore, further studies that consider BMI, hyperandrogenemia, and hyperinsulinemia in women with PCOS that will elucidate the mechanisms underlying mitochondrial dysfunction in the iPSC_PCOS_ model are warranted.

The iPSCs derived from women with PCOS and treated with metformin have been found to be capable of restoring normal biological activity in several DEGs involved in glycogenesis, glucogenesis, and adenosine triphosphate (ATP) generation [164]. On the other hand, in the same study, metformin was found to have a minimal influence on mitochondrial maximal respiration and maximal glycolytic capacity [164]. Indeed, data derived from numerous studies have indicated that treating overweight-obese women with PCOS with metformin reduced their risk for diabetes and CVD, improved their BMI and menstrual irregularity, and normalized their androgen profile [185–188]. The mode of action of metformin is still debated; however, it likely improves mitochondrial respiration through the activation of the AMP-activated protein kinase (AMPK) pathway [189, 190]. According to one recent study, metformin alleviated metabolic derangement, obesity, and ovarian dysfunction in mice with PCOS by regulating the SIRT3/AMPK/mTOR pathway [191].

### Neuroendocrine and Metabolic Characteristics

As discussed earlier, PCOS involves neuroendocrine dysfunction, with Min et al. being the first to publish transcriptome data from iPSC_PCOS_ lines that included neuroendocrine activity and neuronal differentiation [192]. According to their gene enrichment analysis, significantly downregulated DEGs in iPSC_PCOS_ compared to iPSC_Ctrl_ were linked to neurogenesis, enteroendocrine cell differentiation, and the low-density lipoprotein (LDL) particle-binding mechanism. In contrast, neural crest cell growth, the progesterone receptor (PR) signaling pathway, and cholesterol storage mechanisms were found to be associated with the upregulated DEGs. Moreover, the neurotransmitter gamma-aminobutyric acid (GABA) receptor, the cytochrome P450 (CYP) family, the tumor growth factor (TGF)-β pathway, and estrogen receptor (ER)-associated DEGs (*FBP1, PYGL, GAPDH, KDM1A, STAT5, GPI*, and *UGP2*) were found to be linked with neuroendocrine function in their analysis.

Consistent with previous data from Min et al. [164], DEGs linked to glucose metabolism, such as *FBP1, PYGL, GAPDH, GPI*, and *UGP2*, were abnormally expressed, indicating dysregulation of glucose metabolism in iPSC_PCOS_ [192]. Interestingly, neuronal stem cells (NSCs) differentiated from iPSC_PCOS_ showed decreased mitochondrial respiratory capacity consistent with findings in PCOS-derived undifferentiated iPSCs and primary GCs. Furthermore, iPSC_PCOS_ had significantly higher testosterone (T) levels than iPSC_Ctrl_, indicating the potential presence of clinical HA already in the developmental stage [192]. These findings support the theory that a hyperandrogenic intrauterine environment plays a key role in altered ovarian steroidogenesis, insulin metabolism, gonadotrophin secretion, and ovarian follicle formation in PCOS, resulting in typical symptoms in adulthood [193, 194].

## Limitations of the Existing iPSC_PCOS_ Research

To date, hPSC research on women with PCOS has provided promising insights into the early development and progression of PCOS pathogenesis. The findings also confirmed that the PCOS disease model can be produced from any pluripotent cell type, including iPSCs and hESCs obtained from blastocysts. However, thus far, few studies have used hESC_PCOS_ or iPSC_PCOS_, and those that have lacked adequate controls to assess intra-human variability. Moreover, the data that have been generated from hPSC_PCOS_ have been based on only the Rotterdam diagnostic criteria, rather than on the categorization of multivariate independent risk variables, such as obesity, HA, and hyperinsulinemia.

As shown in Table 2, the initial research on hESC_PCOS_ involved heterogeneous phenotypes, had an inadequate sample size for both the disease and control groups, and generated inconsistent findings, calling into question the feasibility of this approach. In addition, disease models derived from hESC lines raise serious ethical and practical concerns. In contrast, one of the key advantages of iPSC_PCOS_ is that it can be readily delivered from somatic cells, thereby increasing the likelihood of acquiring an adequate number of patient-derived iPSC lines, possibly with disease-specific genetic and epigenetic backgrounds. Although iPSCs resemble hESCs both morphologically and functionally, there are several fundamental differences between them, each having significant implications for disease modeling, particularly for hereditary genetic disorders, for which it may not always be possible to replace hESCs with iPSCs [195, 196]. Since this is not the case with PCOS, hPSCs-derived from blastocysts and somatic cells from women with PCOS could be beneficial for modeling the disease, as shown in prior studies.

Concerning the development of an iPSC_PCOS_ model, the risk of partial or complete loss of epigenetic memory upon reprogramming is an important consideration, as it plays a crucial role in determining cell identity, fate, and function [197]. Research utilizing animal and human models has shown that iPSC clones created using distinct cell types from a single donor can effectively dedifferentiate into the same lineage in early passages by retaining the epigenetic memory of the original cells, but appear to lose this memory in late passages [198, 199]. One approach that can be used to determine whether this scenario applies to women with PCOS involves comparing iPSC_PCOS_ with their hESC_PCOS_ counterparts from the same patient, as doing so may reveal the extent to which iPSC reprogramming resets or retains disease-specific epigenetic markers of PCOS. This approach can thus generate insights into the fidelity of reprogramming and can be employed to assess the extent of the epigenetic alterations that occur during the establishment of pluripotent cell lines. However, it must be noted that creating hESC and iPSC lines from a single PCOS patient is not entirely feasible, as, in the donated blastocysts of PCOS patients, half of the female genetic material is replaced by male genetic material.

In addition, the efficiency of the in vitro differentiation (IVD) protocols employed in these experiments is another issue potentially in need of improvement. For example, the GC differentiation protocol [200] used by Huang produced a low yield of GC-like cells, and the upregulation of key markers of GCs seemed fairly low, therefore requiring further optimization. Furthermore, the selection of somatic cells for epigenetic memory-related reprogramming is another topic worthy of investigation. Kajiwara et al. supported the idea that the genetic background of donors is a significant factor in determining the suitability of iPSC clones for IVD, as they determined that variations in their differentiation protocol were largely attributable to donor-based differences rather than to cell origins when comparing iPSCs from peripheral blood and dermal fibroblasts from the same individuals [173, 201]. Moreover, donor age also appears to have a substantial impact on the preservation of genetic and epigenetic memory, as both can be diminished via the use of late passage cells [202]. These issues have not been discussed in the existing hPSC_PCOS_ studies.

## Diagnostic and Screening Challenges for Creating iPSC_PCOS_ Lines

There is clear evidence of racial and ethnic disparities in PCOS [203, 204]. Furthermore, the prevalence of PCOS is affected by both demographic factors and diagnostic criteria. The 2018 international PCOS guideline recommended updating the Rotterdam criteria with both HA and oligomenorrhea (OA) for adolescents based on an evidence-informed expert consensus [8]. Despite this, the Rotterdam criteria are still frequently used for PCOS diagnoses in adults. However, Tay et al. compared the prevalence of PCOS using updated and original Rotterdam criteria in community-based adolescents. According to their findings, the updated 2018 Rotterdam criteria, which include both HA and OA, can identify adolescents at risk for obesity, a critical factor contributing to the severity of PCOS, suggesting that this group should be the focus of early lifestyle interventions and prevention [205].

As discussed earlier, genetic factors play a significant role in predisposing women to PCOS through a combination of direct and indirect gene–environment effects. Moreover, there is evidence to suggest that the intrauterine condition affects fetal PCOS risk, while environmental and lifestyle factors, such as diet, encountered later in life can also play a key role in PCOS risk in adulthood. Thus, it is possible that susceptibility to this disease will be eliminated when generating iPSC lines screened only from clinically diagnosed PCOS by somatic cell reprogramming. On the other hand, theoretically, susceptibility may remain unchanged if patients are screened based on their inherited genetic susceptibility to PCOS by assessing their polygenic risk score (PRS), in addition to the clinical diagnostic criteria. Indeed, the PRS, which was developed from robust GWAS, has been shown to be a potential biological risk predictor for patient stratification and disease risk prediction [206–208]. In connection with PCOS, Joo et al. reported that the PRS for PCOS can be used not only to assess those at increased risk for PCOS but also to detect the wide expression of co-occurring or pleiotropic phenomena associated with PCOS in clinical settings in Europe, Africa, and in many different pedigree participants [209]. Interestingly, another PRS study conducted among first-degree male relatives of PCOS patients showed an increased risk of cardiometabolic and androgenic disorders. This study suggested that genetic risk factors for PCOS may act independently of ovarian function and may have phenotypic effects in men [210].

In the context of creating an iPSC_PCOS_ model, PRS-based screening might represent an additional confirmatory tool to identify well-stratified, genetically predisposed women in addition to the clinical diagnostic criteria [211]. Thus, researchers can identify genetic variables that contribute to the pathogenesis of such a complex syndrome during its development and their transmission between generations, thereby learning more about the etiology of PCOS. However, there are certain limitations to PRS-based screening while creating iPSC_PCOS_ lines that must be considered before introducing a PRS model in PCOS research. As discussed earlier, the current understanding of PCOS-related genetic variants is still evolving; therefore, PRS may not capture the full genetic complexity of these women. Furthermore, as the PRS is a population-level risk assessment tool, a relatively large cohort should be considered to avoid having less predictive power and to enable greater precision.

## From Challenges to Opportunity: Unlocking the Potential of iPSC_PCOS_ Research

Notwithstanding the benefits of iPSC technology, several factors should be taken into account [212]. To draw relevant conclusions about iPSC lines, the standardized procedures used to conduct quality control testing for their characterization must be evaluated. First, the effect of reprogramming methodologies, including viral vector-based methods (retro/lentiviral), non-integrating methods (episomal vectors), and DNA-free methods (RNA/protein) must be taken into account in terms of reprogramming efficiency, genomic integration, safety, and the preservation of disease-specific epigenetic memory. For example, although viral vector-based methods often exhibit higher efficiency, concerns have been raised regarding their effect on genomic integration and the efficiency of transgene silencing [213]. Second, the pluripotency of each cell line should be thoroughly tested (gene expression profile and teratoma formation assay). Moreover, genome integrity and stability using single nucleotide polymorphism (SNP array or DNA sequencing), as well as authentication using short tandem repeat (STR) analysis should be performed to characterize iPSC lines before conducting functional assays [214]. This is because these standardized procedures help to ensure the quality and fidelity of patient-derived iPSC lines and minimize the risk of introducing confounding factors or variability during experimental studies.

Improved, validated, and reproducible IVD protocols may have to be established to determine which among them most reliably mimics the in vivo development of specific cell types and hence most reliably reveals the disease mechanisms. To ensure consistent and reliable differentiation of iPSCs into desired disease-specific cell types, the robustness of IVD protocols should be prioritized [215]. Achieving such robustness involves the characterization of differentiated cells through their physiological, functional, and molecular properties compared to the corresponding in vivo references as a control. Importantly, when considering the disease relevance and epigenetic profiling of the differentiated cells, it is critical to ensure that disease-specific phenotypes are altered or maintained compared to the primary cells. It is also important to employ a large sample size, particularly larger disease groups, to avoid potential inter-individual variations. Validating an IVD model typically also involves integrating multiple cell lines through, for instance, molecular characterization, functional assays, comparisons with reference standards, and correlations with clinical data. By addressing all of these issues, researchers can more effectively evaluate the IVD systems for iPSC_PCOS_ -models and consequently enhance our knowledge of PCOS and its underlying mechanisms.

The iPSC_PCOS_ disease model introduces new methods for illuminating the pathological aspects of metabolic dysregulation in PCOS. Patient-derived iPSC lines can be used for modeling disease mechanisms by differentiating the cells in vitro into affected cell types, followed by GWAS, expression quantitative trait loci (eQTL), and whole genome sequencing (WGS) to identify PCOS pathogenic variants. In addition to monolayer cultures with optimized growth factor cocktails, more advanced 3D organoid and organ-on-a-chip technologies can be employed for PCOS disease modeling [216]. For example, since women with PCOS present with an altered endometrial milieu, it will be of great value to investigate the altered steroid profile, extent of chronic inflammation, and functional metabolomics in iPSC_PCOS_-derived endometrial organoids [217]. Furthermore, women who are resistant to P4 and decidualization for embryo implantation may greatly benefit from the novel insights gained by Cheung et al. concerning P4-responsive endometrial stromal cells using iPSC technology [122]. Similarly, iPSC_PCOS_-derived organoids targeting neuronal cell types (for neuroendocrine disorders) [218], islet cells (hyperinsulinemia-related metabolic dysfunction) [219], adipose tissue (obesity-related metabolic dysfunction) [220], and ovaries (altered steroid metabolism) [221] may yield significant, even groundbreaking, insights by unraveling the etiology of PCOS. Interestingly, these organoid platforms can be employed in genetic manipulation, enabling the investigation of certain genetic alterations and their influence on the development of disease [222]. However, the choice between focusing on conventional 2D cultures and animal models vs. controllable IVD models depends on the specific research goals, available resources, and current understanding of the disease stage. To date, a significant number of studies, including those involving animals, have already been performed, offering a more holistic representation of PCOS pathophysiology, including hormonal regulation and tissue-specific responses. However, laboratory animal models are difficult to manipulate, and the translation of the findings may require additional validation, as they do not accurately reflect human physiology. For instance, in the context of the endometrium, it is extremely challenging to study endometrial regeneration, decidualization, and embryo-maternal interaction in terms of disease progression in vivo due to ethical and practical limitations [223]. In contrast, hPSC_PCOS_-derived IVD models could allow researchers to focus on cell-specific molecular pathways with reproducibility, scalability, and high-throughput experiments relevant to disease pathobiology. Although these IVD models may not capture the full complexity of PCOS, they can broaden our knowledge of the pathogenesis of PCOS. More concisely, iPSC_PCOS_ can be deployed as a tool to conduct basic molecular and functional studies, perform precision therapy, and initiate future drug development, screening, and validation.

## Conclusions and Future Perspectives

Although it is undeniable that hPSC research has enhanced our knowledge of PCOS and laid the groundwork for future investigations of the disease by demonstrating its utility as a model for studying any complex disease, its application to PCOS disease modeling is still in its infancy. In the context of iPSC_PCOS_ research, several factors should be considered before establishing the model. First, general donor-related variability must be considered, particularly when studying heterogeneous PCOS groups (e.g., lean vs. obese, young vs. old, hyperinsulinemia vs. non-hyperinsulinemia, and normo- vs. hyper-androgenemia). The magnitude of donor effects must be thoroughly explored and addressed to avoid discrepancies in final outcomes outside of those related to technical aspects. Besides, in our opinion, focusing on PCOS cases with a high PRS might minimize the influence of noisy environmental factors, as these women are highly likely to develop PCOS regardless of their environmental exposure and epigenetic regulation. In addition, one of the key challenges of such modeling is the lack of available samples, which makes it more difficult to provide corresponding patient-derived primary cells as a control. While non-PCOS iPSCs can be used as controls, they may not present a full picture from which differentiated, cell-derived functional studies can draw firm conclusions. Moreover, an adequate differentiation study might be even more challenging in the context of a multifaceted disease like PCOS. As a result, much work remains to be done to improve the quality and consistency of these outcomes. Consequently, a quantitative assessment of the final quality of cells is required, as is screening for any genetic or epigenetic changes during the reprogramming process.

## Data Availability

Not applicable.

## Declarations

### Search method

The review was written after a thorough search of the literature using the terms “human embryonic stem cells,“ “human induced pluripotent stem cells,“ “polycystic ovarian syndrome”, “genome wide association study”, “polygenic risk score” in the PubMed database. Only articles written in English were included.
