# Correction to: Induced Pluripotent Stem Cells as a Possible Approach for Exploring the Pathophysiology of Polycystic Ovary Syndrome (PCOS)

**DOI:** 10.1007/s12015-023-10663-6

**Published:** 2023-12-23

**Authors:** Masuma Khatun, Karolina Lundin, Florence Naillat, Liisa Loog, Ulla Saarela, Timo Tuuri, Andres Salumets, Terhi T. Piltonen, Juha S. Tapanainen

**Affiliations:** 1 Department of Obstetrics and Gynecology, University of Helsinki, Helsinki University Central Hospital, Haartmaninkatu 8, 00029 HUS Helsinki, Finland; 2 Faculty of Biochemistry and Molecular Medicine, University of Oulu, Oulu, Finland; 3 Institute of Genomics, University of Tartu, 51010 Tartu, Estonia; 4 Department of Genetics, University of Cambridge, Cambridge, CB2 3EH UK; 5 Department of Obstetrics and Gynecology, Research Unit of Clinical Medicine, Medical Research Center, Oulu University Hospital, University of Oulu, Oulu, Finland; 6 Department of Obstetrics and Gynecology, Institute of Clinical Medicine, University of Tartu, 50406 Tartu, Estonia; 7 Competence Centre of Health Technologies, 50411 Tartu, Estonia; 8 Division of Obstetrics and Gynecology, Department of Clinical Science, Intervention and Technology, Karolinska Institutet and Karolinska University Hospital, 14186 Huddinge, Stockholm Sweden; 9 Department of Obstetrics and Gynecology, HFR - Cantonal Hospital of Fribourg and University of Fribourg, Fribourg, Switzerland

**Correction to: Stem Cell Reviews and Reports**

10.1007/s12015-023-10627-w

The Graphical Abstract was missing from this article.

The original article has been corrected.

